# Comparison of short-term outcomes of open, laparoscopic, and robotic surgery for Kasai portoenterostomy in biliary atresia: a 10-year single center study

**DOI:** 10.3389/fsurg.2026.1789037

**Published:** 2026-04-20

**Authors:** Yuliang Jiang, Menglei Zhu, Jianlei Chen, Zhenwei Zhu, Haowei Zhao, Qi Wang, Ziang Wang, Jie Zhu, Peng Cai

**Affiliations:** Department of General Surgery, Children’s Hospital of Soochow University, Suzhou, Jiangsu, China

**Keywords:** biliary atresia, Kasai portoenterostomy, laparoscopy, perioperative outcomes, robotic-assisted surgery

## Abstract

**Objective:**

This study aimed to clarify the comparative clinical efficacy and safety of open (OKPE), laparoscopic (LKPE), and robotic-assisted (RAKPE) approaches for Kasai portoenterostomy in patients with biliary atresia (BA).

**Methods:**

We retrospectively analyzed 50 patients diagnosed with type III BA who underwent Kasai portoenterostomy between January 2015 and December 2024. Based on the surgical approach, patients were categorized into three groups: OKPE (*n* = 21), LKPE (*n* = 18), and RAKPE (*n* = 11). Clinical characteristics, perioperative indicators, and short-term outcomes, including jaundice clearance (JC) at 6 months and one-year survival with native liver (SNL), were compared among the groups.

**Results:**

RAKPE was associated with a significantly longer operative time compared to LKPE and OKPE (310 ± 39 vs. 230 ± 34 vs. 200 ± 74 min; *P* < 0.001). OKPE showed shorter fibrous cone dissection time (47 ± 11 vs. 66 ± 7 vs. 66 ± 9 min; *P* < 0.001) and less dissection blood loss [2(2–3) vs. 4(3–5) vs. 3(2–4) mL; *P* < 0.001]. Conversely, minimally invasive approaches achieved faster oral feeding [10 (8–10) vs. 4.5 (4–5) vs. 4 (4–5) days; *p* < 0.001] and shorter hospital stay [29 (23–36) vs. 19 (15–27) vs. 18 (17–28) days; *P* = 0.003]. No significant differences were observed across the three groups regarding 6-month JC rates (67% vs. 61% vs. 55%; *P* = 0.81), postoperative cholangitis incidence (55% vs. 44% vs. 38%; *P* = 0.66), or one-year SNL rates (71% vs. 72% vs. 64%; *P* = 0.85).

**Conclusions:**

OKPE, LKPE, and RAKPE demonstrate comparable short-term efficacy and safety for type III BA. While OKPE offers technical advantages in hilar dissection, minimally invasive approaches significantly optimize postoperative recovery. Surgical technique selection should be individualized based on patient characteristics, surgeon experience, and institutional resources.

## Highlights

- RAKPE, LKPE, and OKPE show comparable jaundice clearance and native liver survival.
- Minimally invasive approaches significantly accelerate oral feeding and recovery.
- OKPE maintains technical advantages in reducing hilar dissection time and blood loss.
- Robotic-assisted Kasai is safe but currently involves longer operative durations.

## Introduction

Biliary atresia (BA) is a severe neonatal obstructive cholangiopathy characterized by progressive inflammation, fibrosis of intrahepatic and extrahepatic bile ducts (1). Without timely intervention, the patients inevitably progress to end-stage liver cirrhosis and typically die within the first two years of life (2). Since its introduction, the Kasai portoenterostomy (KPE) has remained the cornerstone of surgical management for BA, significantly prolonging survival by reconstructing bile drainage, and transforming the disease from a fatal condition into a manageable chronic disorder (3).

Minimally invasive surgical techniques are expanding the therapeutic landscape for biliary atresia. Laparoscopic Kasai portoenterostomy (LKPE), in particular, has gained increasing acceptance due to its advantages of reduced surgical trauma, faster postoperative recovery, and superior cosmetic outcomes (4, 5). Previous studies have shown that laparoscopic surgery is associated with shorter hospital stays and reduced postoperative pain (6). Robotic-assisted surgery may offer additional technical advantages, including enhanced precision, three-dimensional visualization, and flexible maneuverability (7, 8). However, laparoscopic approaches remain controversial, largely due to the technical challenges of achieving adequate hilar exposure, securing effective hemostasis, and mastering its steep learning curve (9). To date, robotic-assisted Kasai portoenterostomy (RAKPE) remains in an exploratory stage, with limited clinical evidence reported (10, 11). Therefore,this study aims to compare the efficacy and safety of OKPE, LKPE, and RAKPE in the treatment of BA, with the goal of providing evidence-based guidance for optimizing surgical strategies.

## Materials and methods

### Patient selection

Clinical data were retrospectively collected from children with type III BA who underwent KPE at Children's Hospital of Soochow University between January 2015 and December 2024. Inclusion criteria were as follows (1): a confirmed diagnosis of BA based on clinical presentation and intraoperative cholangiography; (2) surgery performed between 50 and 90 days after birth; (3) a follow-up duration of more than 12 months with complete clinical data available. Exclusion criteria included: (1) the presence of other serious congenital anomalies; (2) failure to undergo KPE; (3) loss to follow-up.

After detailed explanation of advantages and disadvantages of each surgical approach, legal guardians independently selected the surgical approach. All surgeries were performed by experienced hepatobiliary surgeons who had completed at least 20 LKPE and RAKPE procedures. Patients were divided into three groups according to the surgical approach: OKPE, LKPE, and RAKPE. No significant differences in baseline characteristics were observed among the three groups (Table 1). This study was approved by the Medical Ethics Committee (Approval No. 2025CS405). Informed consent was obtained from all guardians.

**Table 1:** Baseline characteristics of the patients in OKPE, LKPE, and RAKPE groups.

	Surgical Procedure			
Characteristic	OKPE	LKPE	RAKPE	*p*-value
	*N* = 21	*N* = 18	*N* = 11	
Gender, *n* (%)				>0.99
Female	12 (57%)	11 (61%)	6 (55%)	
Male	9 (43%)	7 (39%)	5 (45%)	
Age at surgery (days), Mean ± SD	61 ± 10	57 ± 11	60 ± 10	0.46
Body weight (kg), Mean ± SD	5.1 ± 0.6	5.0 ± 0.6	5.3 ± 0.9	0.54
Pre-TB (μmol/L), Median (Q1, Q3)	180 (160, 190)	170 (150, 180)	170 (120, 180)	0.45
Pre-DB (μmol/L), Median (Q1, Q3)	130 (110, 150)	120 (110, 140)	110 (87, 140)	0.47
Pre-AST (U/L), Median (Q1, Q3)	270 (200, 300)	210 (170, 270))	180 (150, 280)	0.20
Pre-ALT (U/L), Median (Q1, Q3)	220 (140, 300)	180 (140, 270)	160 (98, 290)	0.43
Fisher's exact test				
One-way analysis of means				
Kruskal–Wallis rank sum test				

Continuous variables are expressed as mean ± standard deviation (SD) or median with interquartile range (Q1, Q3), as appropriate. Categorical variables are presented as frequencies and percentages (*n*, %). TB, Total Bilirubin; DB, Direct Bilirubin; AST, Aspartate Aminotransferase; ALT, Alanine Aminotransferase. Statistical significance among the three groups was assessed using Fisher's exact test for categorical variables, and One-way analysis of variance (ANOVA) or the Kruskal–Wallis rank-sum test for continuous variables.

### Surgical techniques

OKPE: Following laparoscopic biliary exploration, the procedure was converted to an open approach through a right subcostal incision. Step 1: following division of the perihepatic ligaments and liver retraction, the fibrous plaque at the porta hepatis was identified. Dissection continued towards the porta hepatis until reaching the superior edge of the bifurcation of the left and right portal vein branches. The fibrous plate was excised flush with the liver surface, extending bilaterally to the points where the vascular sheaths enter the liver parenchyma. Adequate bile drainage was observed, and meticulous hemostasis was achieved using hemostatic gauze packing. Step 2: the jejunum was identified 25 cm distal to the ligament of Treitz and transected. An end-to-side anastomosis was constructed between the proximal limb and distal bowel loop 40 cm from its closed end. The Roux limb was routed anterior to the duodenum and posterior to the transverse colon, and its open end anastomosed to the porta hepatis.

**Figure 1:**
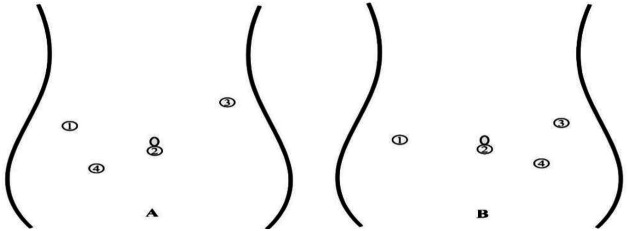
Schematic illustration of port placements. **(A)** Laparoscopic Kasai portoenterostomy (LKPE) group: A 5-mm umbilical trocar is utilized for the laparoscope, accompanied by three 3-mm working trocars placed strategically in the upper and lower abdomen. **(B)** Robotic-assisted Kasai portoenterostomy (RAKPE) group: An 8-mm umbilical trocar is used for the robotic camera, with two 8-mm working trocars (mid-abdominal) for robotic arms, and one 3-mm assistant trocar placed in the left lower quadrant to optimize the operative field.

LKPE: After intraoperative cholangiography confirmation (Figure 1A), a 5-mm umbilical trocar and three 3-mm trocars were placed, and pneumoperitoneum was established. The falciform ligament was retracted to expose the porta hepatis. In accordance with OKPE Step 1, blunt and sharp dissection of the fibrous tissue at the porta hepatis was performed exclusively with a laparoscopic Maryland dissector and microscissors, without electrocautery. Construction of the Roux-en-Y limb and portoenterostomy followed the same principles as OKPE Step 2.

**Figure 2:**
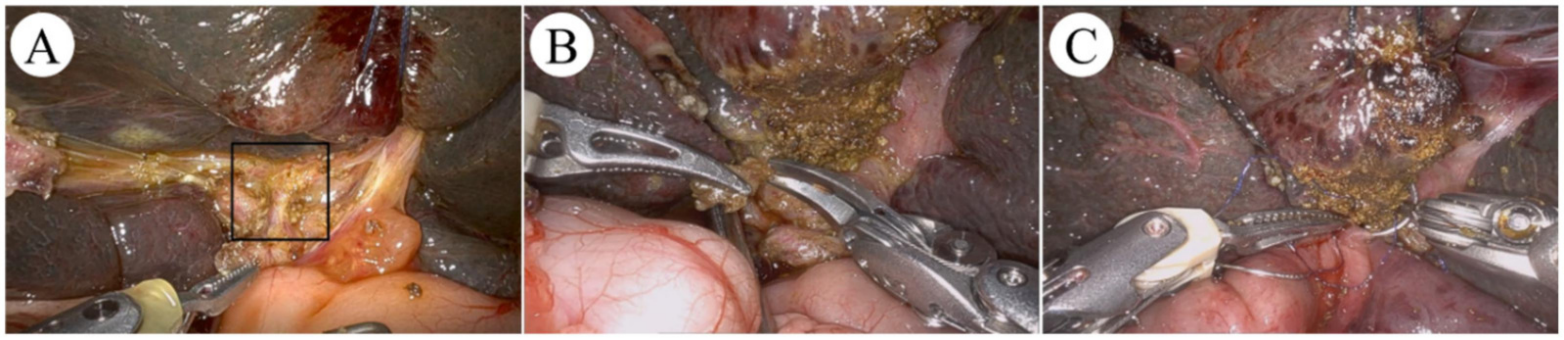
Intraoperative images of the RAKPE group. **A:** Exposure of the hilar fibrous mass; **B:** Trimming of the hilar fibrous mass; **C:** Portoenterostomy.

RAKPE: Following cholangiography confirmation, OKPE Step 2 was performed initially. After bowel replacement, an 8-mm umbilical trocar, two 8-mm mid-abdominal trocars (5.5 cm from umbilicus), and one 3-mm in the left lower quadrant trocar were inserted (Figure 1B). Following reinsufflation, the falciform ligament was retracted, exposing the porta hepatis and clearly revealing the fibrous plate (Figure 2A). In strict accordance with the principle of “cold dissection”, robotic-assisted dissection of the fibrous tissue at the porta hepatis was meticulously performed from left to right using robotic Maryland bipolar forceps and robotic curved scissors. Following careful transection at the bilateral margins, satisfactory bile drainage was observed, with a thin layer of whitish membranous tissue preserved at the resection site (Figure 2B). The procedure was completed successfully, with adequate exposure of the porta hepatis and patent bile drainage channels achieved (Figure 2C).

Postoperatively, intravenous cefoperazone-sulbactam was administered for 2 weeks, followed by oral cefdinir. Intravenous prednisolone (4 mg·kg^−1^·d^−1^) was initiated on postoperative day 5, then switched to oral dosing and tapered over 12 weeks (12).

### Data collection

Collected data encompassed: (1) baseline demographics and clinical characteristics: including gender, age, weight, preoperative liver function parameters (total bilirubin [TB], direct bilirubin[DB], aspartate aminotransferase [AST], and alanine aminotransferase [ALT]); (2) perioperative indicators: including operative time, fibrous plate dissection time, intraoperative blood loss, time to oral feeding, and length of hospital stay; (3) postoperative outcomes, including liver function recovery, postoperative complications, incidence of cholangitis incidence, jaundice clearance rates (JCR, defined as TB < 20 μmol/L) at 1 and 6 months, and one-year native liver survival (NLS) rates (13, 14).

### Statistical analysis

Categorical variables were compared among the three groups using Fisher's exact test, as appropriate. Continuous variables were assessed for normality and are presented as mean ± standard deviation or median with interquartile range (IQR), as appropriate. Comparisons among the three groups were performed using one-way analysis of variance or the Kruskal–Wallis test. *post hoc* pairwise comparisons were conducted using the Steel–Dwass test. All statistical analyses were performed using JMP® Pro version 17.0 (JMP, Cary, NC, USA). A two-sided *p* value < 0.05 was considered statistically significant.

## Results

### Baseline characteristics

Fifty patients were included: OKPE (*n* = 21), LKPE (*n* = 18), RAKPE (*n* = 11). Baseline characteristics were comparable among the three groups (Table 1). Gender distribution showed no significant difference (*p* > 0.99), with females comprising 57%, 61%, and 55% in OKPE, LKPE, and RAKPE groups, respectively. Mean age at surgery did not differ significantly between the OKPE, LKPE, and RAKPE groups (61 ± 10 days vs. 57 ± 11 days vs. 60 ± 10; *p* = 0.46). Body weight averaged 5.1 ± 0.6 kg, 5.0 ± 0.6 kg, and 5.3 ± 0.9 kg for OKPE, LKPE and RAKPE groups, respectively (*p* = 0.54). Preoperative laboratory parameters (TB, DB, AST, ALT) also showed no significant differences (all *p* > 0.05), indicating comparable baseline hepatic status.

### Operative metrics

**Figure 3:**
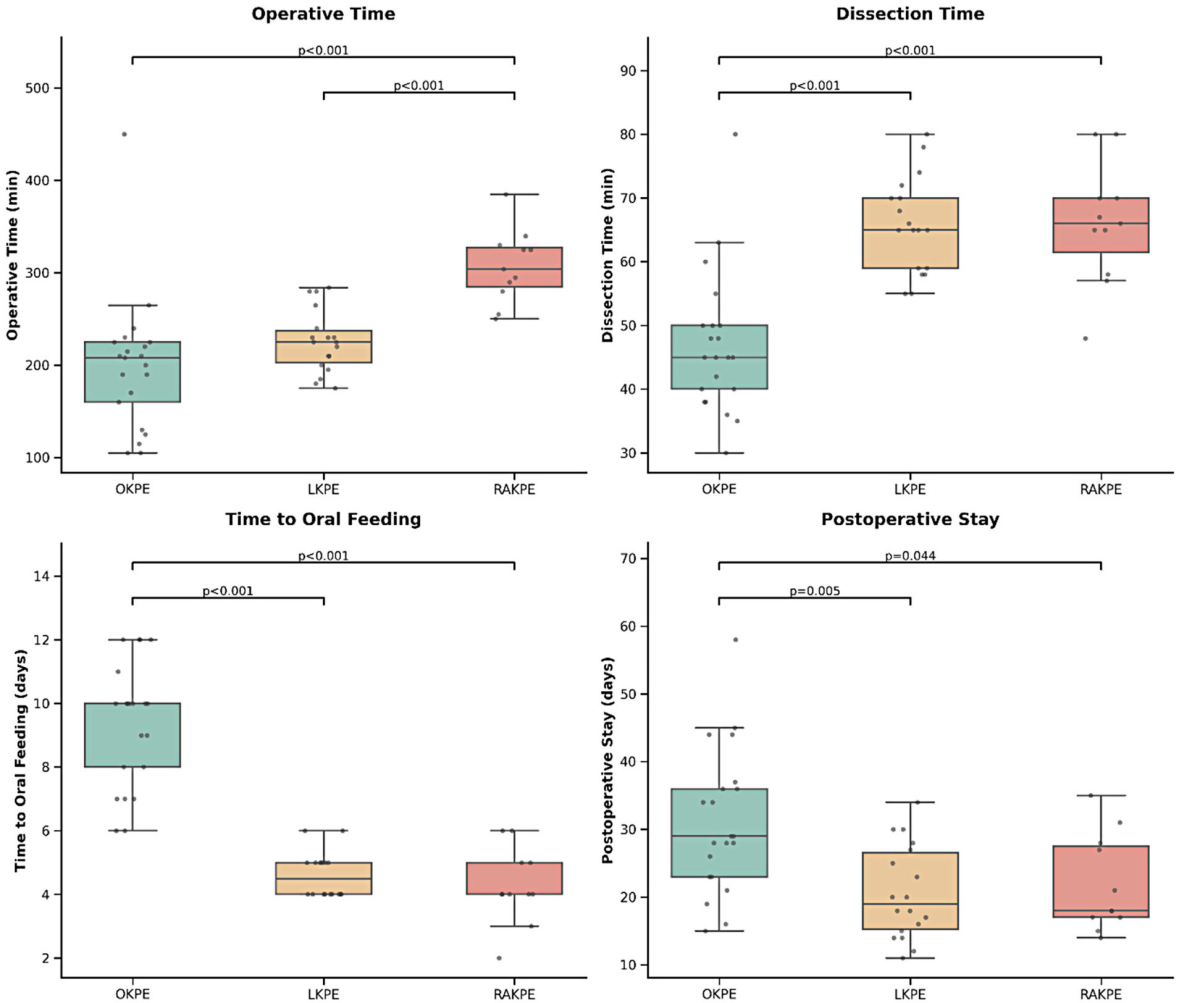
Pairwise comparisons of key perioperative metrics among the OKPE, LKPE, and RAKPE groups. **(A)** Operative time (min), **(B)** Fibrous cone dissection time (min), **(C)** Time to oral feeding (days), and **(D)** Postoperative stay (days). The horizontal line within each box represents the median, while the lower and upper boundaries of the box indicate the first (Q1) and third (Q3) quartiles, respectively. Individual patient data points are superimposed on the boxes (gray dots). *P*-values derived from the *post-hoc* Steel-Dwass test are displayed with brackets to indicate statistically significant differences between specific groups.

Significant differences were observed in operative metrics (Table 2 and Figure 3). Mean operative time was significantly longer in RAKPE group (310 ± 39 min) compared to LKPE (230 ± 34 min) and OKPE (200 ± 74 min) groups (*p* < 0.001) (9, 10). Fibrous cone dissection time was substantially shorter in OKPE (47 ± 11 min) compared to LKPE (66 ± 7 min) and RAKPE (66 ± 9 min) (*p* < 0.001). Total blood loss showed no significant difference (*p* = 0.25), with median values of 20 mL (IQR:15–20), 13 mL (IQR:10–20), and 20 mL (IQR:10–20) for OKPE, LKPE, and RAKPE, respectively. However, blood loss during fibrous cone dissection was significantly lower in OKPE [2 mL (IQR:2–3)] compared to LKPE [4 mL (IQR:3–5)] and RAKPE [3 mL (IQR:2–4)] (*p* < 0.001).

**Table 2:** Comparison of intraoperative metrics and short-term postoperative outcomes among the three surgical groups.

	Surgical Procedure			
Characteristic	OKPE	LKPE	RAKPE	*p*-value
	*N* = 21	*N* = 18	*N* = 11	
Operative time (min), Mean ± SD	200 ± 74^a^	230 ± 34^a^	310 ± 39^b^	<0.001
Fibrous cone dissection time (min), Mean ± SD	47 ± 11^a^	66 ± 7^b^	66 ± 9^b^	<0.001
Total blood loss (mL), Median (Q1, Q3)	20 (15, 20)	13 (10, 20)	20 (10, 20)	0.25
Blood loss during dissection (mL), Median (Q1, Q3)	2 (2, 3)^a^	4 (3, 5)^b^	3 (2, 4)^ab^	<0.001
Time to oral feeding (days), Median (Q1, Q3)	10 (8, 10)^a^	4.5 (4, 5)^b^	4 (4, 5)^b^	<0.001
Post-TB (μmol/L), Median (Q1, Q3)	120 (110, 130)	120 (100, 130)	100 (70, 130)	0.55
Post-DB (μmol/L), Median (Q1, Q3)	96 (81, 110)	92 (75, 99)	90 (77, 100)	0.98
Post-AST (U/L), Median (Q1, Q3)	160 (120, 180)	140 (120, 170)	150 (110, 190)	0.64
Post-ALT (U/L), Median (Q1, Q3)	170 (110, 210)	140 (110, 180)	140 (89, 180)	0.75
Postoperative stay (days), Median (Q1, Q3)	29 (23, 36)^a^	19 (15, 27)^b^	18 (17, 28)^b^	0.003
Postoperative complications (0/1), *n* (%)	3 (14%)	2 (11%)	1 (9%)	>0.99
Wound infection (*n*, %)	2 (9%)	1 (5.5%)	0 (0%)	
Pancreatic leak (*n*, %)	0 (0%)	0 (0%)	0 (0%)	
Anastomotic bleeding (*n*, %)	0 (0%)	0 (0%)	0 (0%)	
Adhesive intestinal obstruction (*n*, %)	1 (5%)	0 (0%)	0 (0%)	
Biliary leakage (*n*, %)	0 (0%)	1 (5.5%)	0 (0%)	
Incisional hernia (*n*, %)	0 (0%)	0 (0%)	1 (9%)	
Cholangitis, *n* (%)				0.66
No	13 (62%)	10 (56%)	5 (46%)	
Yes	8 (38%)	8 (44%)	6 (54%)	
Jaundice clearance at 1 month, *n* (%)				0.87
No	8 (38%)	8 (44%)	5 (45%)	
Yes	13 (62%)	10 (56%)	6 (55%)	
Jaundice clearance within 6 months, *n* (%)				0.81
No	7 (33%)	7 (39%)	5 (45%)	
Yes	14 (67%)	11 (61%)	6 (55%)	
1-year native liver survival, *n* (%)				0.85
No	6 (29%)	5 (28%)	4 (36%)	
Yes	15 (71%)	13 (72%)	7 (64%)	
One-way analysis of means				
Kruskal–Wallis rank sum test				
Fisher's exact test				

Continuous variables are presented as mean ± SD or median (Q1, Q3). Categorical variables are expressed as *n* (%). Superscript letters (a, b) indicate the results of *post-hoc* pairwise comparisons using the Steel-Dwass test; values within a row sharing the same superscript letter are not significantly different, whereas those with different letters are statistically significant (*P* < 0.05). TB, Total Bilirubin; DB, Direct Bilirubin; AST, Aspartate Aminotransferase; ALT, Alanine Aminotransferase; OKPE, Open Kasai Portoenterostomy; LKPE, Laparoscopic Kasai Portoenterostomy; RAKPE, Robotic-Assisted Kasai Portoenterostomy.

### Postoperative recovery

Time to oral feeding was significantly longer in the OKPE group [10 days (IQR:8–10)] compared to LKPE [4.5 days (IQR:4–5)] and RAKPE groups[4 days (IQR:4–5)] (*p* < 0.001). Postoperative laboratory values (TB, DB, AST, ALT) showed no significant differences (all *p* > 0.05), suggesting comparable early hepatic recovery. Hospital stay was significantly longer in OKPE [29 days (IQR:23–36)] compared to LKPE [19 days (IQR:15–27)] and RAKPE [18 days (IQR:17–28)] (*p* = 0.003) (Table 2).

### Complications and outcomes

The overall incidence of postoperative complications was low and comparable among the three groups: 14% (OKPE), 11% (LKPE), and 9% (RAKPE) (*p* > 0.99). Postoperative complications included 2 cases of wound infection and 1 case of adhesive intestinal obstruction in the OKPE group; 1 case of wound infection and 1 case of biliary leakage in the LKPE group; and 1 case of incisional hernia in the RAKPE group. Postoperative cholangitis rates were 38%, 44%, and 55% in OKPE, LKPE, and RAKPE groups, respectively (*p* = 0.66). JCR at 1 month were similar between the OKPE, LKPE, and RAKPE groups: 62%, 56%, and 55% (*p* = 0.87). At 6 months, rates remained comparable: 67%, 61%, and 55% (*p* = 0.81) (14). One-year NLS rates showed no significant difference: 71%, 72%, and 64% (*p* = 0.85).

## Discussion

BA remains one of the most challenging conditions in pediatric hepatobiliary surgery, necessitating prompt intervention to prevent progression to end-stage liver disease (1, 2). Since Kasai's pioneering hepatoportoenterostomy in 1957, KPE has been established as the primary palliative procedure for restoring bile drainage and delaying liver transplantation (3, 15). This study compares perioperative outcomes and safety profiles of three surgical approaches in treating type III BA.

In this cohort, OKPE and LKPE were associated with significantly shorter operative times compared to RAKPE, but no significant difference was observed between OKPE and LKPE The prolonged operative time observed in the RAKPE group is likely attributable to the well-documented learning curve inherent to robotic surgery in pediatric populations (16, 17), including the technical complexity of hilar dissection and additional time required for robotic system setup. Nevertheless, with accumulated surgical experience, standardized protocols, and simulation-based training, operative time reductions of 30–40 min are achievable, potentially approximating LKPE durations after 15–20 cases (18, 19). OKPE exhibits superior performance in fibrous plate dissection time and intraoperative blood loss during hilar dissection (*p* < 0.001). This advantage likely reflect the benefits of direct tactile feedback and unrestricted instrument maneuverability, which prove particularly valuable when managing dense fibrotic tissue at the porta hepatis. RAKPE showed less intraoperative blood loss during hilar plate dissection than the LKPE group, benefiting from the enhanced magnification and tremor filtration of the robotic surgical platform. The development of minimally invasive KPE has been characterized by initial controversy and gradual acceptance. Following Esteves et al.'s first LKPE report in 2002 (4), safety concerns prompted opposition from the International Pediatric Endosurgery Group in 2007. However, subsequent studies, including a recent randomized controlled trial and prospective cohort analyses, have since reported comparable long-term outcomes between LKPE and OKPE (5, 6). Robotic assistance, first reported by Dutta and Albanese (2007) and Meehan et al. (16, 17), confers theoretical advantages that address the inherent limitations of conventional laparoscopy. The da Vinci system delivers seven degrees of freedom with wristed instrumentation, tremor filtration, and high-definition three-dimensional visualization, thereby enhancing precision during delicate hilar dissection and Roux-en-Y reconstruction (11, 20). Although challenges persist—including difficulty in adequate porta hepatis exposure, risk of incomplete fibrous plate excision, hemostatic complications with potential thermal injury, and a steep learning curves—minimally invasive approaches consistently yield faster postoperative feeding and shorter hospital stays, attributable to reduced surgical trauma, diminished pain, and accelerated recovery *p* < 0.001) (6, 21).

Importantly, all three surgical approaches demonstrated comparable short-term outcomes, with no significant differences in JC or 1-year NLS. JC, defined as normalization of serum bilirubin within 6 months post-KPE, represents the most reliable indicator of successful bile drainage and serves as a robust predictor of long-term outcomes (22, 23). Post-KPE cholangitis constitutes the most common serious complication, with reported incidence ranging from 40% to 93% (24). The present study demonstrated comparable rates across all groups (38%–54%), with no significant differences detected. Putative pathophysiologic mechanisms include bacterial translocation, lymphatic dysfunction, portal venous dissemination, and immune-mediated responses. Cholangitis occurring within the first postoperative year adversely impacts outcomes and NLS, serving as a predictor of liver failure and earlier transplantation requirement (25). The RAKPE group exhibited numerically higher cholangitis rates, although this difference did not reach statistical significance, suggesting that achieving technical proficiency requires substantial experience and an extended learning curve. During this developmental phase, RAKPE may not be universally applicable, particularly in patients presenting with severe disease or complex hilar anatomy. For pediatric patients with BA, KPE remains the cornerstone of management, with liver transplantation reserved as salvage therapy. A successful KPE has been shown to confer a significant advantage on subsequent graft survival (26, 27).

This study has several limitations, including a relatively small sample size, single-center design with inherent selection bias, and insufficient long-term follow-up. Future investigations should expand sample sizes through multicenter collaborations, with emphasis on long-term outcome tracking to elucidate differences in cholangitis recurrence, liver fibrosis progression, and transplantation outcomes. Additionally, validating the long-term value of RAKPE and evaluating the cost-effectiveness of robotic technology represent critical research directions. Optimizing surgical techniques, reducing operative times, and minimizing complications through standardized protocols remain ongoing priorities.

## Conclusion

OKPE, LKPE and RAKPE demonstrate comparable short-term efficacy and safety profiles. Surgical technique selection should be individualized based on patient characteristics, surgeon experience, institutional resources, and cost considerations, thereby providing a foundation for personalized treatment planning. As surgical expertise evolves and technology advances, minimally invasive approaches may confer additional benefits while maintaining outcomes equivalent to traditional open surgery.

## Data Availability

The original contributions presented in the study are included in the article/Supplementary Material, further inquiries can be directed to the corresponding author/s.

## References

[1] Hartley JL Davenport M Kelly DA. Biliary atresia. Lancet. (2009) 374(9702):1704–13. 10.1016/S0140-6736(09)60946-6 19914515

[2] Petersen C Davenport M. Aetiology of biliary atresia: what is actually known? Orphanet J Rare Dis. (2013) 8:128. 10.1186/1750-1172-8-128 23987231 PMC3766137

[3] Bezerra JA Wells RG Mack CL Karpen SJ Hoofnagle JH Doo E Biliary atresia: clinical and research challenges for the twenty-first century. Hepatology. (2018) 68(3):1163–73. 10.1002/hep.29905 29604222 PMC6167205

[4] Esteves E Clemente Neto E Ottaiano Neto M Devanir J Esteves Pereira R. Laparoscopic Kasai portoenterostomy for biliary atresia. Pediatr Surg Int. (2002) 18(8):737–40. 10.1007/s00383-002-0791-6 12598978

[5] Son TN Liem NT Hoan VX Tuan KA. Laparoscopic versus open Kasai portoenterostomy for biliary atresia: a randomized controlled trial. J Pediatr Surg. (2023) 58(1):34–9. 10.1016/j.jpedsurg.2022.09.03636283847

[6] Ure BM Kuebler JF Schukfeh N Engelmann C Dingemann J Petersen C. Survival with the native liver after laparoscopic versus conventional Kasai portoenterostomy in infants with biliary atresia: a prospective trial. Ann Surg. (2011) 253(4):826–30. 10.1097/SLA.0b013e318211d7d8 21475026

[7] Sheetz KH Claflin J Dimick JB. Trends in the adoption of robotic surgery for common surgical procedures. JAMA Netw Open. (2020) 3(1):e1918911. 10.1001/jamanetworkopen.2019.18911 31922557 PMC6991252

[8] Moglia A Georgiou K Georgiou E Satava RM Cuschieri A. A systematic review on artificial intelligence in robot-assisted surgery. Int J Surg. (2017) 41:151–60. 10.1016/j.ijsu.2017.03.073 34695601

[9] Cundy TP Harling L Hughes-Hallett A Hughes-Hallett A Mayer EK Najmaldin AS Meta-analysis of robot-assisted vs conventional laparoscopic and open pyeloplasty in children. BJU Int. (2014) 114(4):582–94. 10.1111/bju.12683 25383399

[10] Woo R Le D Albanese CT Kim SS. Robot-assisted laparoscopic resection of a type I choledochal cyst in a child. J Laparoendosc Adv Surg Tech A. (2006) 16(2):179–83. 10.1089/lap.2006.16.179 16646713

[11] Davenport M Ong E Sharif K Alizai N McClean P Hadzic N Biliary atresia in England and Wales: results of centralization and new benchmark. J Pediatr Surg. (2013) 48(7):1543–50. 10.1016/j.jpedsurg.2013.04.013 21929975

[12] Section of Pediatric Hepatic Transplantation, Branch of Organ Transplantation, Chinese Medical Doctor Association. Guidelines for diagnosing & treating biliary atresia. Chin J Pediatr Surg. (2019) 40(5):392–8. 10.3760/cma.j.issn.0253-3006.2019.05.003

[13] Serinet MO Wildhaber BE Broué P Broué P Lachaux A Sarles J Impact of age at Kasai operation on its results in late childhood and adolescence: a rational basis for biliary atresia screening. Pediatrics. (2009) 123(5):1280–6. 10.1542/peds.2008-1949 19403492

[14] Shneider BL Magee JC Karpen SJ Rand EB Narkewicz MR Bass LM Total serum bilirubin within 3 months of hepatoportoenterostomy predicts short-term outcomes in biliary atresia. J Pediatr. (2016) 170:211–217.e2. 10.1016/j.jpeds.2015.11.058 26725209 PMC4826612

[15] Kasai M Kimura S Asakura Y Suzuki H Taira Y Ohashi E. Surgical treatment of biliary atresia. J Pediatr Surg. (1968) 3(6):665–75. 10.1016/0022-3468(68)90897-X

[16] Dutta S Woo R Albanese CT. Minimal access portoenterostomy: advantages and disadvantages of standard laparoscopic and robotic techniques. J Laparoendosc Adv Surg Tech A. (2007) 17(2):258–64. 10.1089/lap.2006.0112 17484663

[17] Meehan JJ Elliott S Sandler A. The robotic approach to complex hepatobiliary anomalies in children: preliminary report. J Pediatr Surg. (2007) 42(12):2110–4. 10.1016/j.jpedsurg.2007.08.040 18082719

[18] Meehan JJ Sandler AD. Pediatric robotic surgery: a single-institutional review of the first 100 consecutive cases. Surg Endosc. (2008) 22(1):177–82. 10.1007/s00464-007-9418-2 17522913

[19] Cundy TP Shetty K Clark J Chang TP Sriskandarajah K Gattas NE The first decade of robotic surgery in children. J Pediatr Surg. (2014) 49(6):858–65. 10.1016/j.jpedsurg.2014.01.006 23583146

[20] Zhang MX Tang JF Zheng ZB Cao G-Q Zhou Y Zhou Y Comparison of surgical results and technical performance between robotic and laparoscopic approaches for Kasai portoenterostomy in biliary atresia: a multicenter retrospective study. Surg Endosc. (2025) 39(2):1128–39. 10.1007/s00464-024-11452-z 39702567

[21] Murase N Hinoki A Shirota C Tomita H Shimojima N Sasaki H Multicenter, retrospective, comparative study of laparoscopic and open Kasai portoenterostomy in children with biliary atresia from Japanese high-volume centers. J Hepatobiliary Pancreat Sci. (2019) 26(2):43–50. 10.1002/jhbp.594 30488647

[22] Davenport M Superina R. Primary liver transplant in biliary atresia: the case for and against. J Pediatr Surg. (2024) 59(7):1418–26. 10.1016/j.jpedsurg.2024.03.005 38565475

[23] Pandurangi S Kim S Asai A Bondoc A Balistreri W Campbell K Customized postoperative therapy improves bile drainage in biliary atresia: a single center preliminary report. J Pediatr Surg. (2023) 58(8):1483–8. 10.1016/j.jpedsurg.2022.10.050 36496264 PMC10846645

[24] Decharun K Leys CM West KW Finnell SM. Prophylactic antibiotics for prevention of cholangitis in patients with biliary atresia status post-Kasai portoenterostomy: a systematic review. Clin Pediatr (Phila). (2016) 55(1):66–72. 10.1177/0009922815594760 26183324

[25] Calinescu AM Madadi-Sanjani O Mack C Schreiber RA Superina R Kelly D Cholangitis definition and treatment after Kasai hepatoportoenterostomy for biliary atresia: a delphi process and international expert panel. J Clin Med. (2022) 11(3):494. 10.3390/jcm11030494 35159946 PMC8836553

[26] Fouquet V Alves A Branchereau S Grabar S Debray D Jacquemin E Long-term outcome of pediatric liver transplantation for biliary atresia: a 10-year follow-up in a single center. Liver Transpl. (2005) 11(2):152–60. 10.1002/lt.20358 15666395

[27] Hsu EK Shaffer ML Gao L Sonnenday C Volk ML Bucuvalas J Analysis of liver offers to pediatric candidates on the transplant wait list. Gastroenterology. (2017) 153(4):988–95. 10.1053/j.gastro.2017.06.053 28711630 PMC6288076

